# An innovative method of nonclinical efficacy and toxicological evaluation of recombinant *Salmonella typhi* Ty21a expressing HPV16 and 18 L1 proteins

**DOI:** 10.1016/j.mex.2021.101219

**Published:** 2021-01-06

**Authors:** Yathapu Srinivasa Reddy, K. Narendra Babu, S.S.Y.H. Qadri, M.V. Surekha, B. Dinesh Kumar

**Affiliations:** a Advanced Centre for Preclinical Toxicology Studies, ICMR-National Institute of Nutrition, Jamai-Osmania, Hyderabad – 500007; b Department of Microbiology and Immunology, ICMR-National Institute of Nutrition, Jamai-Osmania, Hyderabad – 500007; c Animal Facility, ICMR-National Institute of Nutrition, Jamai-Osmania, Hyderabad – 500007; d Department of Pathology, ICMR-National Institute of Nutrition, Jamai-Osmania, Hyderabad - 500007

**Keywords:** Innovative toxicological evaluation, Recombinant *Salmonella typhi* Ty21a vector, HPV 16 and 18 L1 proteins

## Abstract

Advancement in technology led to development of live attenuated *Salmonella typhi* Ty21a as enteric vector for expression of foreign proteins. Such vector platform is inevitable for development of vaccine candidate against human papilloma virus (HPV), the etiological agent of cervical cancer with high prevalence in developing nations. The high risk HPVs like type 16 and 18 contributes to 70% of cervical cancer, hence Indian Immunologicals Limited (IIL), Hyderabad, India developed a recombinant HPV vaccine by introducing HPV 16 and 18 L1 protein coding genes into attenuated *S. typhi* Ty21a vector. Being a genetically engineered enteric vector vaccine, it would be less expensive, with an ease of oral administration, instead of injectable that needs trained personale, is an added advantage for low socioeconomic setup compared to existing HPV vaccines.

Establishing the nonclinical efficacy and safety/toxicity as per the national/international regulatory guidelines has become major constrain for such recombinant *S. typhi* HPV (rSt.HPV) vaccine. Since, the intended clinical mode of rSt.HPV is through oral route, whereas the live attenuated *S. typhi* Ty21a doesn't colonize in gut of laboratory animals to be used for nonclinical experiments. Hence, an alternate and unconventional method of ‘intranasal drug testing’, was followed for nonclinical efficacy and toxicity evaluations. An array of parameters specified by regulatory agencies were investigated in mice, rat and rabbits administered with rSt.HPV through, intra-peritoneal, intranasal and oral routes, the intended clinical route.

• Current unconventional and innovative nonclinical testing procedures helps in exploring the alternate methods by pharmacologist/toxicologist.
• Ultimately, such new drugs developed through technology must serve the humankind justifying the guidelines of regulatory agencies.

## Graphical abstract

**Figure:**
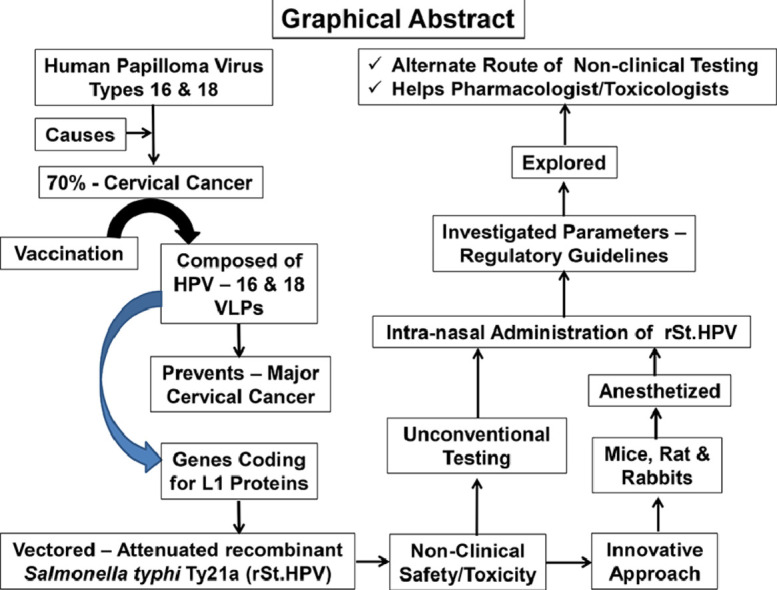

Specifications table

**Table:** 

Subject Area:	Pharmacology, Toxicology and Pharmaceutical Science
More specific subject area:	Non clinical Efficacy and Toxicology Evaluation of Recombinant HPV Vaccine
Method name:	Intranasal Testing
Name and reference of original method:	Nardelli-Haefliger D., et al. 1997. Human papillomavirus type 16 virus-like particles expressed in attenuated Salmonella typhimurium elicit mucosal and systemic neutralizing antibodies in mice. Infect. Immun. 65:3328–3336.
Resource availability:	Not Applicable

## Method Details

### Back ground of method development

Current modified method was originally reported by Nardelli-Haefliger et al. [1] to demonstrate the immune response evaluation against HPV 16 L1 proteins expressed through live attenuated *Salmonella typhimurium* CS022 vector. The *S. typhimurium* was administered to mice through oral and nasal routes. However, an advancement in technology over a period led to fine tune such recombinant vaccines in various aspects viz. selection of live attenuated vectors which colonize in human gut, *in vivo* construct stability, constitutive expression system, markers other than antibiotic resistance etc.. In view of the mentioned properties and the expertise in development of recombinant vaccines, the M/s. Indian Immunologicals Limited (IIL), Hyderabad, India has developed a proprietary anti-HPV vaccine candidate by introducing the codon optimized HPV 16 and 18 L1 protein coding genes into live attenuated *Salmonella typhi* Ty21a as vector, as the HPV 16 and 18 together responsible for 70% of cervical cancers caused throughout the world [2], [3], [4].

The commercially available vaccines against HPV are expensive, being the injectable needed the cold-chain system and trained personale for administration. In addition these vaccines were known to have the adverse effects such as headache, gastrointestinal disturbances, syncope, myalgia, fever, etc. Whereas, the recombinant *S. typhi* Ty21a expressing HPV 16 and 18 L1 proteins (r.St.HPV) developed by IIL, Hyderabad, India has advantages over existing vaccines in terms of its cost-effectiveness, admissible through oral route hence doesn't required sophisticated infrastructure thus better suits for low-socioeconomic countries. It is noteworthy to mention here that a higher prevalence of HPV related cervical cancers were reported from developing and low-socio economic countries with a mortality rate of 88% [5].

The current innovative method confines for establishing the nonclinical efficacy and safety/toxicity as per the national/international regulatory guidelines. The major constrain is the inability of live attenuated *S. typhi* Ty21a colonization in gut of laboratory animals when administered through oral route, which is the intended clinical route of vaccination, hence nonclinical efficacy and safety/toxicity studies of r.St.HPV has become challenging [6]. Hence, an alternate and unconventional method of ‘intranasal drug testing’, was followed for nonclinical efficacy and toxicity evaluations. An array of parameters specified by regulatory agencies were investigated in mice, rat and rabbits, which were administered with rSt.HPV through intra-peritoneal, intranasal and oral routes, the intended clinical route [7].

## Materials and Methods

### Chemicals, Reagents & sterile-ware

1. Ketamine Hydrochloride (obtained with approval of Central Drugs Standard Control Organization (CDSCO) of India and utilized under the Drug/Anesthesia Custodian of Institute), (Bharat parenteral Ltd, Gujarat, India)
2. Xylaxine hydrochloride (Indian Immunologicals Limited, Hyderabad, India)
3. Diazepam (Centaur Pharmaceuticals, Mumbai, India),
4. Water for injection (Deo Gratias Parenteral, MP, India),
5. Graduated sterile tubes (Corning:15mL# CLS430791- Axygen:2.0mL # SCT-200-SS-C-S)
6. Sterile Filtered tips (Axygen: TF-1000-R-S, TF-200-R-S & TF-20-R-S),
7. Sterile Phosphate Buffered Saline (PBS, GIBCO#10010023)
8. Autoclaved and dried bedding husk,
9. Stainless steel trays,
10. Sterile disposable syringes with needles (BD 329654),
11. 70% Isopropyl alcohol (IPA) swabs,
12. Nitrile gloves (Kimberly-Clark # U20306)
13. Salmonella Shigella Agar (SS Agar, HI-Media # M108D)

### Particulars of Laboratory animals

1. Swiss Albino mice, 4–6 weeks young, weighing ~18–20 g;
2. Sprague Dawley rats, 4–6 weeks young, weighing ~150–180 g;
3. New Zealand White rabbits, weighing ~1.5–2.0 kg.

### Source of Laboratory Animals & Approvals

1. Animals were obtained from ‘National Centre for Laboratory Animal Sciences’ (NCLAS), ICMR-National Institute of Nutrition (ICMR-NIN), Hyderabad, India.
2. Experimental animals were handled with standard care and the procedures were approved by ‘Institutional Animal Ethics Committee’ (IAEC) of ICMR-NIN, Hyderabad, India.
3. Since the test compound is a recombinant vaccine, the approvals from Institutional Biosafety Committee (IBSC) and Review Committee on Genetic Manipulation (RCGM) – Department of Biotechnology (DBT), India were also obtained.

### Diet and Water for animals

1. Standard sterile pellet diets comprised of all macro and micronutrients (Tables 1 and 2) according to mice, rat and rabbits requirements were obtained from NCLAS, ICMR-NIN, Hyderabad, India [8].

**Table 1:** Pellet Diet Composition.

S. No.	Name of Ingredient	Percent Composition	
		Mouse / Rat	Rabbit
1.	Wheat flour	22.5 %	61.3 %
2	Roasted Bengal gram flour	60 %	28.2 %
3	Skim Milk Powder	5 %	5 %
4	Casein	4 %	1 %
5	Refined Oil	4 %	5 %
6	Salt Mixture with starch	4 %	4 %
7	Vitamin & Choline mixture with starch	0.5%	0.5 %
8	Vitamin-‘C’	–	50 mg/100 g diet
9	Bengal Gram (Sprouted)	–	20 g
10	Lucerne Grass	–	100 g

**Table 2:** Composition of Salt Mixture and Vitamin Mixture.

Composition of Salt Mixture			Composition of Vitamin Mixture		
S. No.	Name of Mineral	Per 100 kg diet (g)	S. No	Name of Vitamin	Per 100 kg diet (g)
1.	Dicalcium Phosphate (CaHPO_4_)	1250.00	1.	(dl) –α-Tocopherol Acetate 50% Dry Powder (E)	12.0 g
2.	Calcium carbonate (CaCO_3_)	555.00	2.	Menadione (K)	0.15 g
3.	Sodium Chloride (NaCl)	300.00	3.	Thiamine (B1)	1.2 g
4.	Magnesium sulphate (MgSO_4_7H_2_0)	229.20	4.	Riboflavin (B2)	0.5 g
5.	Ferrous Sulphate (FeSO_4_ 7H_2_0)	50.00	5.	Pyridoxine (B6)	0.6 g
6.	Manganese sulphate (MnSO_4_.H_2_0)	16.04	6.	Niacin	1.0 g
7.	Potassium Iodide (KI)	1.00	7.	Pantothenic Acid (Calcium Salt)	1.2 g
8.	Zinc sulphate (ZnS0_4_ 7H_2_0)	2.192	8.	Cyanocobalamine (B12)	0.5 µg
9.	Copper sulphate (CuSO_4_.5H_2_0)	1.908	9.	Folic Acid	0.1 g
10.	Cobalt Chloride (CoCl_2_.6H_2_0)	0.012	10.	Para amino Benzoic Acid (PABA)	10.0 g
Weight of all Minerals together (g)		2405.35	11.	Biotin	40.0 mg
Starch to be added (g)		1594.65	12.	Inositol	10.0 g
Total Weight (g)		4000.00	13.	Choline Chloride	100.0 g
i.e. 4.0kg for 100 kg of Diet			Weight of all vitamins together (g)		176.76
			Starch to be added (g)		323.24
			Total Weight (g)		500.00
			i.e. 500 g of vitamin mixture is used for every 100 kg of the diet prepared
2. In addition to standard pellet diet, rabbits were given ‘Lucerne grasses’ and Sprouted Bengal gram [8].
3. Diet and purified water collected through activated charcoal filter and subsequently exposed to UV light were given as *ad libitum* to animals.

### Test compound details

The following test compounds were produced and supplied by the IIL, Hyderabad, India. The lyophilized powder of test compound was confirmed for potency in terms of viable count, expression of 55 kDa L1 protein antigen through immunoblot, plasmid stability etc. following good manufacturing practices (GMP) and the same was provided as certificate of analysis by IIL, Hyderabad, India. The intended clinical mode of vaccine administration is through oral route with a dose of 2 × 10^9^ CFU/70 kg adult human.

1. Sterile phosphate buffer saline (PBS),
2. Lyophilized powder of live attenuated *Salmonella typhi* Ty21a,
3. Lyophilized powder of recombinant live attenuated *S. typhi* Ty21a with HPV 16 & 18 L1 protein coding genes.

### Test compound dosing

**Table:** 

Vector	: Live attenuated *Salmonella typhi* Ty21a
Adult human dose / clinical prophylactic dose (PD)	: 2 × 10^9^ CFU of *S. typhi* Ty21a with HPV/70 kg
Intended clinical route of application	: Oral
Form of test compound administration to humans	: Suspension of bacterial vector possessing HPV
Non-clinical testing	: Acute, Sub-chronic, Allergenic and immunogenicity
Route of Non-clinical testing	: Intra-peritoneal, Intra-nasal and oral routes
Prophylactic dose for non-clinical testing	: [Pharmacological Conversion factor]x[Human PD]
Mice non-clinical PD/kg	: [0.13] x [2 × 10^9^] = 0.26 × 10^9^ CFU/kg mice.
Rat non-clinical PD/kg	: [0.09] x [2 × 10^9^] = 0.18 × 10^9^ CFU/kg Rat.
Rabbit non-clinical PD/kg	: [0.045] x [2 × 10^9^] = 0.09 × 10^9^ CFU/kg Rabbit.

#### Acute Toxicity Testing Doses

**Table:** 

Mice non-clinical acute dose		
(i) 10xPD		: [10] x [Mice PD]
		: [10] x [0.26 × 10^9^] = 2.6 × 10^9^ CFU/kg mice
(ii) 50xPD		: [50] x [Mice PD]
		: [50] x [0.26 × 10^9^] = 13 × 10^9^ CFU/kg mice
Rat non-clinical acute dose		
(i) 10xPD		: [10] x [Rat PD]
		: [10] x [0.18 × 10^9^] = 1.8 × 10^9^ CFU/kg rat
(ii) 50xPD		: [50] x [Rat PD]
		: [50] x [0.18 × 10^9^] = 9.0 × 10^9^ CFU/kg rat

#### Sub-chronic Toxicity Testing Dose

#####  

**Table:** 

Rat non-clinical sub-chronic dose	
(i) Prophylactic dose for non-clinical testing (PD)	: [1] x [Rat PD]
	: [1] x [0.18 × 10^9^] = [0.18 × 10^9^] CFU/kg Rat.
(ii) Average dose (AD) for non-clinical testing	: [5] x [Rat PD]
	: [5] x [0.18 × 10^9^] = 0.9 × 10^9^ CFU/kg Rat.
Rabbit non-clinical sub-chronic dose	
(i) Prophylactic dose for non-clinical testing (PD)	: [1] x [Rabbit PD]
	: [1] x [0.09 × 10^9^] = 0.09 × 10^9^ CFU/kg Rabbit.
(ii) Average dose (AD) for non-clinical testing	: [5] x [Rabbit PD]
	: [5] x [0.09 × 10^9^] = 0.45 × 10^9^ CFU/kg Rabbit.

#### Allergenic and Immunogenicity Testing Dose for Mice

#####  

**Table:** 

Intra-peritoneal (IP) Allergenic and Immunogenicity Testing	
(i) Prophylactic dose for IP- Allergenic and Immunogenicity	: [1] x [PD]
	: [1] x [0.50 × 10^5^] = 0.50 × 10^5^ CFU/kg mice
(ii) Average dose for IP- Allergenic and Immunogenicity	: [5] x [PD]
	: [5] x [0.50 × 0^5^] = 2.50 × 10^5^ CFU/kg mice
Intra-nasal (IN) Allergenic and Immunogenicity Testing	
(i) Prophylactic dose for IN- Allergenic and Immunogenicity	: [1] x [PD]
	: [1] x [0.26 × 10^9^] = 0.26 × 10^9^ CFU/kg mice
(ii) Average dose for IN- Allergenic and Immunogenicity	: [5] x [PD]
	: [5] x [0.26 × 10^9^] = 1.30 × 10^9^ CFU/kg mice

### Procedure

#### Preparation & Administration of Anastasia

1. The ingredients of ‘anesthesia’, their role and composition were given in Table 3.

**Table 3:** Anesthesia Composition:.

S. No.	Name of Chemical	Role of Ingredient	Composition (mg/B.Wt.)
1.	Ketamine Hydrochloride	Acts as dissociative general anesthetic (animal not fully unconscious) with high therapeutic index (wide dose range)	62.5 mg/kg
2.	Xylaxine hydrochloride	Sedative property with mild analgesic action. Not induce surgical depth of anesthesia as alone.	1.0 mg/kg
3.	Diazepam	Fat-soluble mild sedative, calm and relax the animal.	1.0 mg/kg
2. Ketamine hydrochloride was collected into sterile graduated tube and then added the Xylaxine hydrochloride, followed by gentle mixing of the contents.
3. The Diazepam was added to Ketamine-Xylaxine mixture.
4. Anesthesia solution was mixed gently and brought the final concentration through addition of sterile distilled water.
5. The tubes were labelled and placed in Ice pack gels, until administration.
6. Body weight of animals to be anesthetized were recorded and the anesthesia collected accordingly into ‘1.0 mL’ sterile syringes.
7. Animal (mice & rat) brought to ‘supine position’ by holding tail of ‘prone position’ animal with one hand and dorsal-neck skin with other hand, followed by tilting the animal 180^o^ across the plane.
8. The lower quadrant of abdominal area (fur shaved region) was whipped with 70% IPA swabs, and subsequently allowed to dry.
9. A volume of (75–100 µL / mice and 750–1000 µL / rat) of anesthesia solution was administered to animals according to their body weights through intra-peritoneal route.
10. Delivery of anesthesia was ensured by introducing the syringe into lower abdominal skin with an angle of ~10°, to avoid accidental penetration into viscera.
11. Rabbits were administered 1.5–2.0 mL of anesthesia through intramuscular route (fur shaved region of hind limb thigh, whiped with 70% IPA) according to body weight.
12. The unconscious stage of anesthetized animals were achieved at 5–10 min of post-exposure of anesthesia and persist for 20–30 min.
13. Anesthetized animals were laid-down in supine position with their heads raised using bedding husk (Fig. 1A, 2A & 3A).

**Fig. 1:**
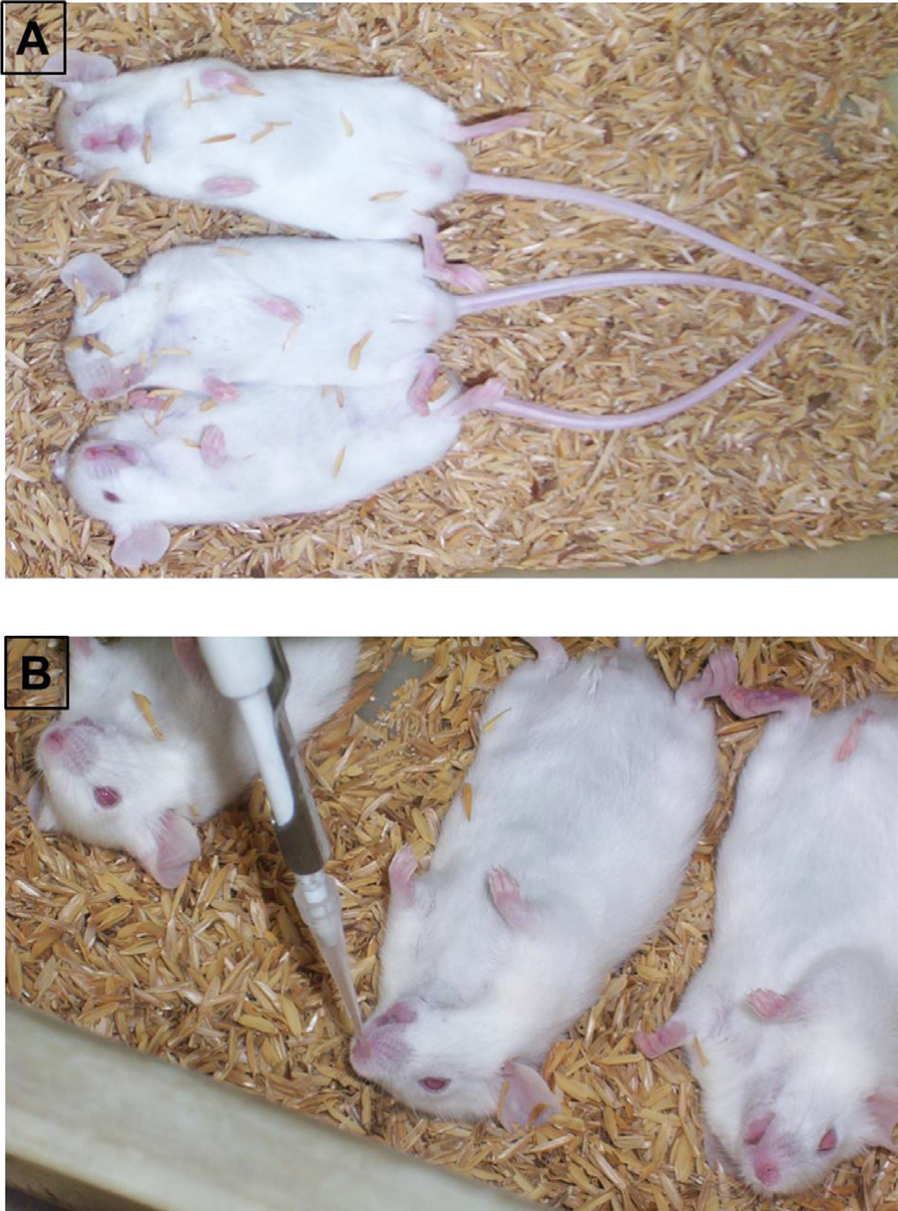
Test compound administration to mice. A: Anesthetized mice. B: Intra-nasal vaccine administration to anesthetized mice.

#### Test Compound Suspension Preparation

1. Lyophilized powder of attenuated *S. typhi* Ty21a and recombinant *S. typhi* Ty21a expressing HPV 16 and 18 L1 proteins (rSt.HPV) were obtained from Indian Immunologicals Limited (IIL), Hyderabad, India.
2. The lyophilized powders of *S. typhi* and rSt.HPV were diluted in sterile PBS under aseptic conditions.
3. Bacterial concentrations of suspension were determined by plating three successive dilutions in duplicate on Salmonella-Shigella agar.
4. The bacterial colonies were enumerated after incubation at 37°C and expressed as ‘colony forming units’ (CFU) /mL.
5. Stock bacterial suspensions were prepared according to previous step and diluted to highest doses of 50xPD, 10xPD, AD (5xPD) and PD of mice, rat and rabbits.

#### Bacterial suspension Administration

**Fig. 2:**
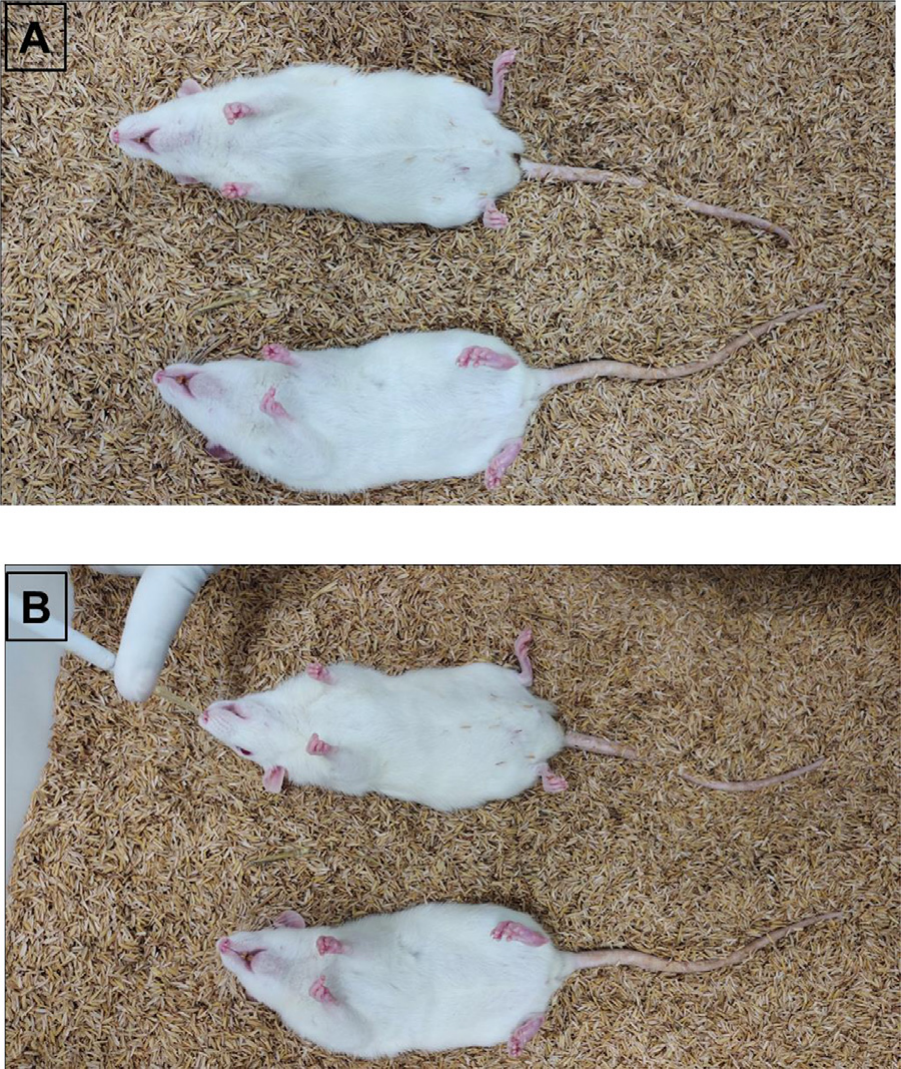
Test compound administration to rat. A: Anesthetized rat. B: Intra-nasal vaccine administration to anesthetized rat.

1. The acute testing of live attenuated *S. typhi* Ty21a expressing HPV 16 and 18 L1 proteins was performed in mice and rats by single administration of 10xPD and 50xPD through intranasal route.
2. Sterile filtered tip fitted micro-pipets were loaded with the designated volumes of bacterial suspension according to the body weights of animals.
3. The volumes not greater than 20 µL and 100 µL were administered to anesthetized unconscious stage, supine positioned mice and rats, respectively through right nostril (Fig. 1, Fig. 2). Whereas, for rabbits a volume of not greater than 200 µL of vaccine was administered through right nostril under anesthetized condition (Fig. 3B).

**Fig. 3:**
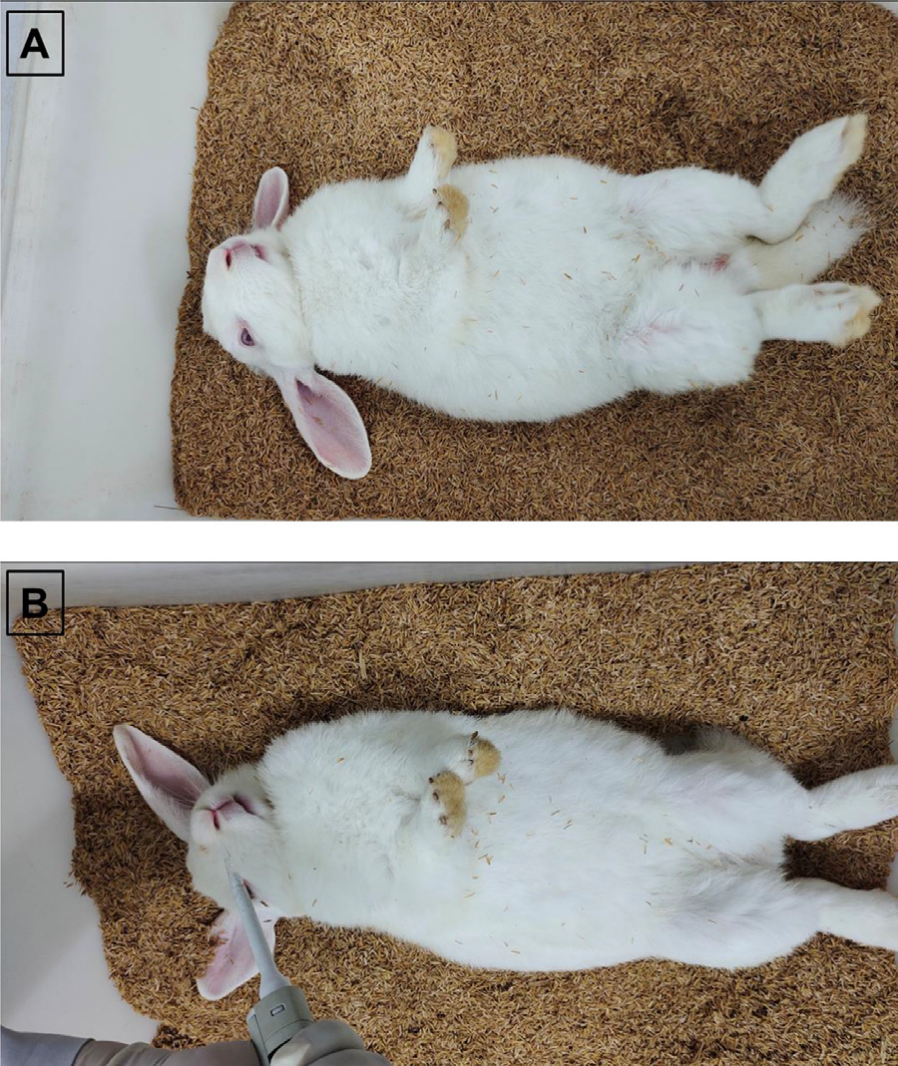
Test compound administration to rabbit. A: Anesthetized rabbit. B: Intra-nasal vaccine administration to anesthetized rabbit.
4. After test compound administration, animals on husk bedding were monitored until they get consciousness, without disturbing.
5. Similarly, the sub-chronic toxicity testing of rSt.HPV vaccine was undertaken in rats and rabbits with three successive dosing on 1^st^, 3^rd^ and 5^th^ day by administration of two concentrations of vaccine i.e., PD and AD (5xPD).
6. In sub-chronic toxicity testing, the prophylactic doses (PD) of vaccine was administered through intranasal routes to anesthetized rats and rabbits. In addition to intranasal route, the prophylactic dose effect of vaccine was assessed through oral route in rats and rabbits.
7. The average dose (AD=5xPD) effect of vaccine was undertaken through intranasal route only in rats and rabbits, as mentioned above.
8. The allergenic and immunogenicity potential of the r.St.HPV vaccine was assessed by administration of test compound through intra-peritoneal (IP) and intra-nasal (IN) routes in mice. Mice were primed with 250 µL of vaccine (PD and AD (5xPD)) by administration through intra-peritoneal route after whipping the local area with 70% IPA. Similarly, the booster dose was administered on 7^th^ day.
9. Intranasal route of allergenic and immunogenicity evaluation was undertaken in mice, anesthetized as explained above. Anesthetized mice were primed with 20 µL of vaccine (PD and AD (5xPD)) administration through IN route on 0^th^ day following the ‘step no. 2–4’. Subsequently the mice were anesthetized and boosted with vaccine on 28^th^ and 56^th^ days through IN route.
10. All the animals were observed and investigated for below mentioned parameters until the end of the experimental period as per the regulatory guidelines.

### Nonclinical Efficacy and Safety Studies Undertaken

#### Acute Toxicity Testing

1. The mice and rat administered with single exposure of 10xPD and 50xPD doses of vaccine were observed for morbidity and pre-terminal mortality on 1^st^, 2^nd^, 3^rd^, 6^th^, 12^th^ and 24^th^ hour of post-vaccine exposure.
2. Later on the animals were observed for 14 days for cage side activities along with recording of bodyweights bi-weekly.
3. The food and water intake was recorded daily.
4. In case of pre-terminal mortality, autopsy was carried out to unveil the test compound effect if any on vital organs, otherwise all the animals were euthanized on 14^th^ day by examining the animals for gross necropsy and collecting the vital organs for archiving.

#### Sub-chronic Toxicity Testing

1. The sub-chronic toxicity testing of vaccine was assessed by repeated exposure of prophylactic and average doses of r.St.HPV on 1^st^, 3^rd^ and 5^th^ day through intranasal and oral routes.
2. The vaccine exposure effect, if any, in relation with duration was evaluated by periodic euthanization of animals at various time points viz., 15^th^ day (1/4^th^ of animals from each group), 29^th^ (another 1/4^th^ of animals) and 93^rd^ days (remaining 50% of animals).
3. The urine, blood, vital organs/tissues and femur bone were collected at respective time points as explained below.

#### Allergenic and Immunogenicity Testing of rSt.HPV

1. The nonclinical efficacy evaluation of test compound i.e. live attenuated recombinant S. typhi Ty21a expressing HPV 16 and 18 L1 protens (rSt.HPV), was undertaken in mice through two different routes.
2. Allergenic profile of vaccine, if any was assessed by administration of Ovalbumin (2% OVA) to mice (positive control) and quantification of specific IgE levels in serum.
3. The PD and AD were administered through intra-peritoneal routes on 0^th^ day and one booster dose was administered on 7^th^ day. Serum anti-HPV 16 and 18 specific IgG and IgE levels were determined on 0^th^, 14^th^ and 28^th^ day. Half of the animals were euthanized on day 14^th^ and remaining on 28^th^ day to unveil the test compound exposure effect on various organs systems.
4. Bacterial suspension of prophylactic dose (PD) and average dose (AD) were administered to anesthetized mice through intra-nasal route as explained under above sections. The animals were primed with test compound on 0^th^ day and subsequently boosted on 28^th^ and 56^th^ days. Blood was collected on 0^th^, 28^th^, 56^th^, 63^rd^ and 78^th^ days to assess anti-HPV 16 and 18 specific IgG and IgE levels. At the end of experiment mice were euthanized and collected the vital organs/tissues for histopathological examination.

### Observations and Parameters Investigated and Analyzed

The animals were observed for morbidity and mortality as per the regulatory guidelines. The following observations, bio-sample collection and analysis of various parameters were undertaken to unveil the ‘vaccine administration effect’, if any.

#### Functional observation battery (Safety pharmacology)

##### Live phase of animals

The animals were observed daily for ‘behavioral activities’ viz., active, non-active, partially active and hyperactive. The water and food intake was monitored daily along with recording of body weights on alternate days.

##### Cage side observations

The vaccine exposed animals were monitored for their home cage activity viz., lying on side, resting, and alertness were monitored daily. The excretion of urine and fecal matter amount, colour and consistency were noted daily. In addition, the behavior of the animals while removing from cage was recorded whether the animals were quiet easily removed / runs around in the cage / oriented towards the investigator/aggressive or any vocalization.

##### Physical Examination

Periodically the animals were observed for physical appearance of hair coat, piloerection, lacrimation, salivation, respiration character and rate, along with eye prominence and eye lid closure. In addition, the biting behavior, convulsions and tremors if any, were examined daily.

##### Neurological Examination

The effect of vaccine administration on neurological functions were assessed in terms of locomotor activity, rearing activity, tail elevation, static limb position, abnormal & ataxic gait, head position and pinna touch response twice in a week.

##### Allergenicity

Animals administered with vaccine were examined daily for the allergenic effect of test compound, if any, in terms of skin reactions and hair loss, along with watering and congestion of eyes.

##### Sleeping time during anesthesia

In order to intranasal administration of vaccine, the animals were anesthetized. Therefore sleeping time during anesthesia were recorded from time of anesthesia administration, sleeping induction time, drug administration time and wake up time.

#### Bio-sample Collection

##### Urine Collection

From all the animals, 24 h urine was collected by placing individual animal in the ‘metabolic cages’, specifically designed for urine collection. These cages were designed in such a way that the animals have access for food and water, in addition the urine neither mixes with feces nor spillover water/feed. Urine collected was qualitatively analyzed using Ames Multistix strips (Siemens).

##### Blood Collection

The effect of test compound administration on various organ systems and metabolism was assessed by collection of blood and investigating the sensitive biomarkers. In view of this, blood was collected from all the animals through retro-orbital plexus using the heparinized capillaries. Briefly, animal was restrained, then the skin of neck gently scruffed, thus the eye made to bulge. Heparinsed capillary tube inserted medially and the blood was collected into tubes that were either pre-coated with anti-coagulants or plain tubes, as aliquots. Then an aliquot of blood samples were subjected for serum/plasma separation by centrifugation.

##### Vital organs/tissue collection

The process of vital organs/tissues that were collected was explained below.

#### Clinical laboratory investigations

##### Qualitative urine analysis

The urobilinogen, protein, pH, RBC, specific gravity, ketone, bilirubin and glucose were analyzed qualitatively in urine samples collected prior to vaccine administration and at different time points of post-exposure using Ames Multistix reagent strips (Siemens).

##### Clinical chemistry

Effect of vaccine administration on various organ systems were assessed by albumin, total protein, calcium and glucose levels along with liver function markers such as alanine aminotransferase (ALT), aspartate aminotransferase (AST) and alkaline phosphatase (ALP) levels in serum samples collected at different time points. In addition, the renal function markers viz., creatinine, total bilirubin and urea levels were also estimated in serum of the animals exposed with vaccine.

##### Hematology

The effect of test compound exposure on blood was investigated in terms of total white blood cell (WBC), red blood cell (RBC), hemoglobin (Hb), hematocrit (HCT), mean corpuscular volume (MCV), mean corpuscular hemoglobin (MCH), mean corpuscular hemoglobin concentration (MCHC), platelet count, mean platelet volume (MPV) and differential leucocytes counts. These investigations were carried out in blood collected in to EDTA K2 vacutainer tubes at different time points.

##### Immunotoxicology

The immunotoxic effect of vaccine exposure if any, was assessed through tier-I and tier-II tests. The tier-I test includes a battery of investigations viz. CBC (Compete blood count), differential blood count, lymphoid organ weights, body weights, histopathology of lymphoid organs, spleen and bone marrow cellularity. When the tire-I tests yield evidence of immune dysfunction, then tire-II tests would be carried out. The tire-II tests include mitogen stimulation of B and T cells, primary (IgM, IgG) humoral response to T-dependent antigen, delayed type hypersensitivity response (DTH) and mouse ear swelling test (MEST).

#### Euthanization and necropsy

At the end of the experimental period the animals were fasted overnight (feed withdrawn and water provided) and blood was collected through retro-orbital plexus and euthanized either through cervical dislocation (mice and rat) or administration of high dose of anesthesia through marginal vein (rabbit). Then the animals were examined for lymphnode enlargement through gross examination process followed by necropsy and collection of vital organs as illustrated below.

##### Gross necropsy examination

The animals were thoroughly examined for any physical external abnormalities. Then the abdominal cavity and chest region opened and carefully observed for multitude of lesions if any, and any macroscopic changes in organs systems.

##### Collection of vital organs

The organs viz., brain, heart, lungs, liver, spleen, kidneys (left & right), gastrointestinal tract, pancreas, individual sex organs, thymus, thyroid, trachea, adrenals, bone marrow and site of injection were collected carefully and once again the individual organs were examined for gross changes. Then the weights of organs were recorded using sensitive balance, subsequently sliced adequately and transferred into 10% neutral buffered saline.

##### Histopathology

The effect of test compound exposure on various organs/tissues were assessed by ‘histological examination’. The organs/tissues were processed by conventional methods after a minimum of 24 h fixation, subsequently paraffin blocks were prepared to obtain 4 µm sections. Then the sections were stained with Hematoxylin and Eosin and examined under a research microscope to unveil the cytological variations, if any. Histological changes in tissues were recorded and compared with corresponding controls.

#### Genotoxicity

The bone marrow micronucleus test was used to evaluate the genotoxic effect of vaccine exposure, if any. The femur bones of all the animals were collected and marrow was flushed using foetal calf serum. A fine colloid of the flushed out marrow was prepared and mounted on the slides after air-drying from the sediment after centrifuging at 800 rpm for 5 min. The staining was done using May-Gruenwald stain and followed by Geimsa stain. The scoring was done for 1000 PCEs and the presence of NCEs and micro nucleated PCEs and the ratio (PCE/NCE) has been calculated.

#### Immunogenicity/Allerginicity Evaluation

1. The immunogenicity potential of rSt.HPV vaccine was evaluated by determining the HPV 16 and 18 L1 specific IgG levels in serum samples of mice administered with test compound.
2. In addition, the allergenic effect, if any of vaccine was assessed through determination of HPV 16 and 18 specific IgE levels in reference to OVA specific IgE levels.
3. The Maxisorp^TM^ plates (96 well, Nunc, Denmark) were coated either with HPV 16 and 18 L1 or OVA proteins in Carbonate-bicarbonate buffer (Sigma #C3041) (100 ng/100 µLwell) by overnight incubation at 4°C.
4. Unbound protein was removed and wells were washed thrice with 300µL phosphate buffer saline containing 0.05% Tween-20 (Sigma # P7949) (PBST).
5. Then, 200 µL of 2% (w/v) skimmed milk in PBST was used to block the remaining active surface of wells by incubation at 37°C/2 h, subsequently wells were washed with PBST.
6. Hundred microliters of diluted anti-sera (for IgG 1:100 and IgE 1:50) collected from mice administered with vaccine and controls were loaded per well and incubated at 37°C/1 h.
7. Wells were washed thrice with 300 µL/well PBST and 1:5000 diluted sheep anti-mouse IgG (A90-141A Bethyl Laboratories) / IgE(A90-115A Bethyl Laboratories)-HRP conjugate was loaded (100 µL / well) and incubated at 37°C/1 h, subsequently the plates were washed thrice with 300 µL/well PBST.
8. The chromogenic reaction was developed by addition of H_2_O_2_ as substrate (100µL/well) and 3,3´,5, 5´ –Tetramethylbenzidine as indicator, followed by incubation in dark at room temperature for 15–30 min.
9 The reaction was terminated by addition of 100 µL of H_2_SO_4_ (0.18 M) / well, followed by measurement of colour intensity at 450 nm using multimode reader.

## Outcome of Method Development

The dossier comprised of nonclinical efficacy and toxicology experimental results obtained from mice, rat and rabbits administered with live attenuated recombinant *Salmonella typhi* Ty21a expressing HPV 16 and 18 L1 proteins were furnished to ‘competent regulatory authorities’. In addition, the same was published in ‘Vaccine Journal’ (DOI: https://doi.org/10.1016/j.vaccine.2020.11.023).

## Declaration of Competing Interest

The authors declare that they have no known competing financial interests or personal relationships that could have appeared to influence the work reported in this paper.
